# The human DNA ends proteome uncovers an unexpected entanglement of functional pathways

**DOI:** 10.1093/nar/gkw121

**Published:** 2016-02-25

**Authors:** Vivien Berthelot, Gildas Mouta-Cardoso, Nadia Hégarat, François Guillonneau, Jean-Christophe François, Carine Giovannangeli, Danièle Praseuth, Filippo Rusconi

**Affiliations:** 1 Laboratoire de chimie physique, UMR CNRS 8000, University of Paris-Sud, F-91400 Orsay, France; 2 Structure et Instabilité des Génomes, INSERM U1154, UMR CNRS/MNHN 7196, F-75005 Paris, France; 3 Plateforme de spectrométrie de masse 3P5, Institut Cochin, F-75014 Paris, France; 4 Inserm and Sorbonne Universities, UPMC, UMR_S 938, Research Center Saint-Antoine, F-75012 Paris, France

## Abstract

DNA ends get exposed in cells upon either normal or dysfunctional cellular processes or molecular events. Telomeres need to be protected by the shelterin complex to avoid junctions occurring between chromosomes while failing topoisomerases or clustered DNA damage processing may produce double-strand breaks, thus requiring swift repair to avoid cell death. The rigorous study of the great many proteins involved in the maintenance of DNA integrity is a challenging task because of the innumerous unspecific electrostatic and/or hydrophobic DNA—protein interactions that arise due to the chemical nature of DNA. We devised a technique that discriminates the proteins recruited specifically at DNA ends from those that bind to DNA because of a generic affinity for the double helix. Our study shows that the DNA ends proteome comprises proteins of an unexpectedly wide functional spectrum, ranging from DNA repair to ribosome biogenesis and cytoskeleton, including novel proteins of undocumented function. A global mapping of the identified proteome on published DNA repair protein networks demonstrated the excellent specificity and functional coverage of our purification technique. Finally, the native nucleoproteic complexes that assembled specifically onto DNA ends were shown to be endowed with a highly efficient DNA repair activity.

## INTRODUCTION

In living cells, the DNA molecule exposes free ends either in the course of normal processes or because of dysfunctional cellular and molecular events. Normal processes include telomeric maintenance (1), V(D)J (2) and meiosis (3,4). Abnormal events include failure during topoisomerase catalytic cycles (5), stalled replication forks (6) and clustered DNA damages induced by either UV radiations (7) or ionizing radiations (8,9). Depending on the cellular and molecular context in which DNA ends get exposed, the requirement to have them sensed, protected or processed, changes. For example, undesirable double-strand breaks, which expose DNA ends, need to be swiftly dealt with by the cell because they may induce cell death if left unrepaired (10).

The wide variety of functional pathways that are committed to the maintenance of DNA integrity involves a very large number of proteins of often critical function. Many of these proteins, when dysfunctional, constitute the etiological factor of pathologies that permitted their discovery. The severe combined immunodeficiency syndrome (SCID) has been at the root of innumerous protein function characterizations, like the co-discovery of the XLF/Cernunnos protein function (11,12).

The cellular response to the presence of DNA ends materializes itself with a finely orchestrated formation of DNA–protein assemblies that can be studied with various strategies. One strategy is based on the preparation of chromatography resins coupled to already described proteins as baits to purify their interactors (13–15). Another strategy involves DNA oligonucleotide-based chromatography resins that mimic DNA structures of interest onto which proteins are expected to bind specifically. This latter strategy has the significant advantage to involve direct interactions between the DNA oligonucleotide and the proteins that specifically bind to it (16–18). Both strategies share serious technical limitations related, for a large part, to the numerous contaminants that bind unspecifically to the chromatographic support and that are released—together with the proteins of interest—upon elution (19,20). In a previous report, we had successfully addressed these limitations by introducing a photocleavable linker between the DNA duplex oligonucleotide and the chromatographic support. The ability to detach the purified nucleoproteic complexes from the beads allowed us both to attain unprecedented protein enrichment levels and to massively reduce contaminating biochemical reactions, such as spurious phosphorylations of the XRCC4 protein by kinases unspecifically bound to the beads (21).

Here, we report the first human DNA ends proteome. Our aim was to specifically isolate proteins onto double-strand DNA ends. Unspecific proteins were removed from the identified proteome thanks to an original control chromatographic procedure. Nucleoproteic complexes specifically assembled onto DNA ends were found to be endowed with a highly efficient DNA repair activity. These complexes were directly visualized by blue native gel electrophoresis (22) and the related proteome was identified by semi-quantitative mass spectrometry. The proteomics data were mined with Free Software specially developed for this project. We find that the DNA ends proteome comprises a moderate set of proteins of an unexpectedly wide functional spectrum, ranging from DNA repair to ribosome biogenesis and cytoskeleton. Further, we define a subset of that proteome made only of proteins either not known to be involved in DNA ends-related processes or of totally undocumented function.

## MATERIALS AND METHODS

All the experiments were performed using nuclear extracts from cultured HeLa non-confluent cells (Ipracell S.A., Belgium). Benzonase was from Merck and DNase I was from Invitrogen. The magnetic streptavidin-coated beads were from Roche. The pUC 19 plasmid (Invitrogen) was used as a template for PCR-based production of biotinylated duplex DNA oligonucleotides. The PCR primers endowed with a photocleavable biotin moiety were from Eurogentec. Colloidal G250 Coomassie blue was from Thermoscientific.

### Home-made chromatographic phase preparation

The forward (GCTGCAAGGCGATTAAGTTGG) and reverse (TGTGGAATTGTGAGCGGATAAC) biotinylated primers (Supplementary Figure S1) were designed to produce duplex DNA oligonucleotides in either a monobiotinylated form (only the forward primer is biotinylated) or a bibiotinylated form (both primers are biotinylated). Following a standard PCR using the relevant primers and pUC 19 as the matrix, the produced material was purified by anion exchange chromatography to get rid of the remaining primers in the PCR mixture. Preparation of the chromatographic phases was typically performed by overnight incubation, at 4°C under gentle rotation, of either 15 pmol or 7.5 pmol of oligonucleotides (respectively mono- or bibiotinylated) with 350 μg of streptavidin-coated beads. The functionalized beads were then magnet-sedimented and washed using the TEN 1000 buffer (10 mM Tris-HCl, 1 mM EDTA, 1 M NaCl).

Checking the proper binding of the biotinylated duplex DNA oligonucleotides on the streptavidin-coated beads was performed by incubating separately each chromatographic phase as prepared above with 20 units of the Sma I restriction enzyme (New England Biolabs, buffer NEB4) for 2 h at 30°C (Supplementary Figure S2).

The data set reported in this article has been obtained by performing six independent monobiotinylated phase-based experiments and five independent bibiotinylated phase-based experiments as detailed in section *Sampling scheme* in the Supplementary Information.

### Affinity-based DNA repair protein purification

A typical purification involved 350 μg of streptavidin-coated beads functionalized as described above and 800 μg of HeLa nuclear extracts (20 mM HEPES; 100 mM KCl; 220 mM glycerol; 0.5 mM PMSF; 0.5 mM DTT; 0.2 mM EDTA; 0.01% NP40) (21). Briefly, the two components were mixed and let incubate at 30°C for 10 min under gentle agitation. The beads were then magnet-sedimented and the pellet was washed four times with 200 μl of the Ku buffer (40 mM HEPES; 5 mM MgCl_2_; 60 mM KCl; 0.4 mM EDTA; 37 mM glycerol; 0.01% NP40; 0.5 mM DTT; pH 7.5).

The chromatographic slurry was then equilibrated in 200 μl of the BN-0 buffer (50 mM bis-Tris; 109 mM glycerol; 1 mM EDTA; pH 7.5). Following magnet-sedimentation of the beads, these were resuspended in 35 μl of the BN-500 buffer (BN-0 supplemented with 500 mM amino caproic acid). The resuspended beads were irradiated for 20 min with a UXM-200HO xenon-mercury lamp (λ > 300 nm, 90 mW/cm^2^ at λ ≈ 365 nm, 5% light transmittance at λ ≈ 295 nm; Lot-Oriel, Palaiseau, France). During the irradiation, the samples were kept chill by laying the container over salty ice. The beads were then pelleted and the supernatant, containing the purified material, was further analyzed. One experiment (see Supplementary Figure S3) allowed us to determine that the energy used in this UV irradiation step was not enough to induce detectable amounts of cyclobutane pyrimidine dimers.

### Footprint assay of the purified nucleoproteic complexes

Radiolabeled DNA was prepared by incubation of dephosphorylated monobiotinylated duplex DNA oligonucleotides with polynucleotide kinase (New England Biolabs) in the presence γ [^32^P]-ATP (PerkinElmer). The resulting DNA was used to functionalize the beads according to the conventional protocol described above.

The purified nucleoproteic complexes were detached from the beads as described above. The supernatant was subjected to DNase I (1.5 unit, Invitrogen) and benzonase (1 unit, Merck) digestion for 1 h at 37°C under gentle agitation. Following the DNA digestion, the remaining nucleoproteic complexes were deposited in two different lanes of a BN-PAGE gel. After the electrophoresis, one lane was stained by colloidal Coomassie blue and further autoradiographed. The unstained lane was then used to excise gel regions corresponding to the bands observed on the autoradiography film (Supplementary Figure S4). The polyacrylamide gel material was then crushed and the contained DNA was water-extracted and purified on Spin-X centrifuge tube filters (Costar). The DNA was sequenced using the Maxam and Gilbert method.

### Ligation assay

Bibiotinylated duplex DNA oligonucleotides (7.5 pmol) were digested using Pst I (New England Biolabs, buffer NEB3). The 108 bp and 78 bp fragments thus produced were used to functionalize the beads as described above. For the ligation assay, 320 μg of nuclear extracts were used for the purification. The purified nucleoproteic complexes were washed with 200 μl of BN-0 buffer and resuspended in 35 μl of this same buffer. During the ligation experiment, the samples were supplemented with T4 DNA ligase buffer (New England Biolabs), 2.6% of PEG 8000, 5% glycerol and 7.5 pmol of the same 108 bp and 78 bp fragments as above (previously UV-irradiated so as to remove the biotin moiety, thus freeing their 5'P end). Ligation assays were performed under two schemes: either the nucleoproteic complexes tested were still attached to the beads or they were previously detached by UV irradiation. Ligations were carried over at 30°C for 2 h under gentle agitation. Control experiments were performed by heating the nuclear extracts at 80°C for 5 min prior to the ligation step. After the ligation step, the proteins bound to the DNA were treated for 1 h at 55°C with 1 mg/ml Proteinase K (New England Biolabs) in 20 mM Tris-HCl supplemented with 0.5% SDS and 10 mM EDTA. The remaining nucleic acids material was further deproteinized by phenol/chlorophorm/isoamyl alcohol extraction followed by ethanol precipitation and analyzed by 2% agarose gel electrophoresis followed by ethidium bromide staining. A control ligation assay was performed by incubating the total nuclear extracts with the exact same amount of DNA as for the experiments above but with no chromatographic support. This experiment was performed 3 times with consistent results.

### Native polyacrylamide gel electrophoresis

The purified nucleoproteic complexes were deposited onto a NativePAGE gel (Life technologies) and migrated according to the Blue Native PAGE technique (22–24). Anode buffer: 500 mM bis-Tris-HCl pH 7. Cathode buffers: 150 mM bis-Tris-HCl, pH 7; 500 mM Tricine, supplemented with either 2.4 mM G-250 Coomassie blue (dark cathode buffer) or 0.24 mM G-250 Coomassie blue (light cathode buffer).

The migration is first performed for 1 h at 100 V with the dark cathode buffer. The dark buffer is replaced by the light buffer and the migration is continued at 200 V until the migration front reaches the bottom of the gel.

The blue-colored gels were incubated in a 30% ethanol:10% acetic acid solution in water overnight at room temperature under gentle agitation. Rehydration of the destained gels was performed with two washes in a 20% ethanol solution followed by two washes in bidistilled water for further staining with either colloidal Coomassie blue (25) or silver stain (26).

### Mass spectrometry

Stained bands were excised from the gels and subjected to in-gel trypsic digestion using standard procedures. Analyses were performed on an Ultimate 3000 Rapid Separation liquid chromatographic system (Dionex, The Netherlands) coupled to a hybrid LTQ-ORBITRAP ‘Velos’ mass spectrometer (Thermo Fisher Scientific, San José, CA, USA). Briefly: acidified peptides were loaded and washed on a C_18_ reversed phase precolumn (3 μm particle size, 100 Å pore size, 75 μm column internal diameter, 2 cm column length) using a loading buffer containing 98% water, 2% acetonitrile and 0.1% trifluoroacetic acid. The flow rate was 5 μl/min. Peptides were then separated on a C_18_ reversed phase analytical column (2 μm particle size, 100 Å pore size, 75 μm column internal diameter, 15 cm column length) with a 45 min gradient from 99% buffer A (5% acetonitrile in 0.1% formic acid in water) to 40% buffer B (80% acetonitrile in 0.085% formic acid in water).

Data were acquired throughout the elution process by a linear trap quadrupole(LTQ)–orbitrap mass spectrometer operated in a data dependent scheme as follows: full MS scans were acquired with the orbitrap in lock mass internal calibration on the siloxan background ion (m/z 445.12063), followed by up to 10 LTQ MS/MS CID spectra on the most abundant precursors detected in the previous MS scan. Exclusion latency was set to 24 s for previously fragmented precursors. Mass spectrometer settings were, for full MS acquisitions, automatic gain control (AGC) set at 1.106, resolution set at 3.104, mass-to-charge ratio (m/z) range set at 400–2000, maximum ion injection time of 1000 ms; for MS/MS acquisitions, AGC set at 1.104, maximum injection time was 200 ms, minimum signal threshold set at 2000, isolation width of 2 Da, collision energy set at 35% RF amplitude, activation time of 10 ms. Only precursors bearing 2, 3 or 4 positive charges were fragmented.

The raw mass data were converted to XML-formatted data files using the msconvert program of the Proteowizard software suite running in MS-Windows (27). The output format was mzXML, with TPP compatibility enabled and 64-bits binary encoding precision. All the remaining data processing steps were performed on a Debian GNU/Linux platform (http://www.debian.org) using the following set of software programs: the X!Tandem protein identification software that uses tandem mass spectrometry data (28). Full details about the database searching and protein identification and semi-quantitation using emPAI are provided in the Supplementary Information.

An *I* index estimating the specificity of each protein for the free DNA ends was calculated according to the following equation:

\begin{equation*} I = \frac{\displaystyle \sum _{i=1}^{N} \frac{M_i}{M_i+B_i}}{N} \end{equation*}

where *M*_*i*_ and *B*_*i*_ are the emPAI values of the protein purified respectively on the DNA ends phase (Monobiotinylated) and the control phase (Bibiotinylated); *N* is the number of experiments in which that protein was identified in the whole set of six experiments. Only the proteins identified in at least 3 experiments over the 6 were considered (i.e. *N* ≥ 3).

## RESULTS

### Crafting of a specific affinity-chromatography system

To identify the subpopulation of nuclear proteins specifically recognizing and binding to DNA ends, two affinity chromatography phases were designed, exposing or not DNA ends. The DNA ends-exposing phase was meant to purify nucleoproteic complexes specifically assembling onto DNA ends while the other was used as a control phase recruiting any protein presenting a generic affinity for duplex DNA. The two phases were thus referred-to below as the ‘DNA ends phase’ and the ‘control phase’. The comparison of the proteins purified on the two phases allowed us to resolve the set of proteins specific for the DNA ends. The PCR-based chromatographic phase design is described in Supplementary Figure S1 and the preparation procedure is described in *Materials and Methods*. The control experiment demonstrating that the bibiotinylated duplex DNA did indeed bind to the chromatographic support *via* both of its biotin moieties, thus producing affinity determinants lacking DNA ends, is described in Supplementary Figure S2.

**Figure 1.**
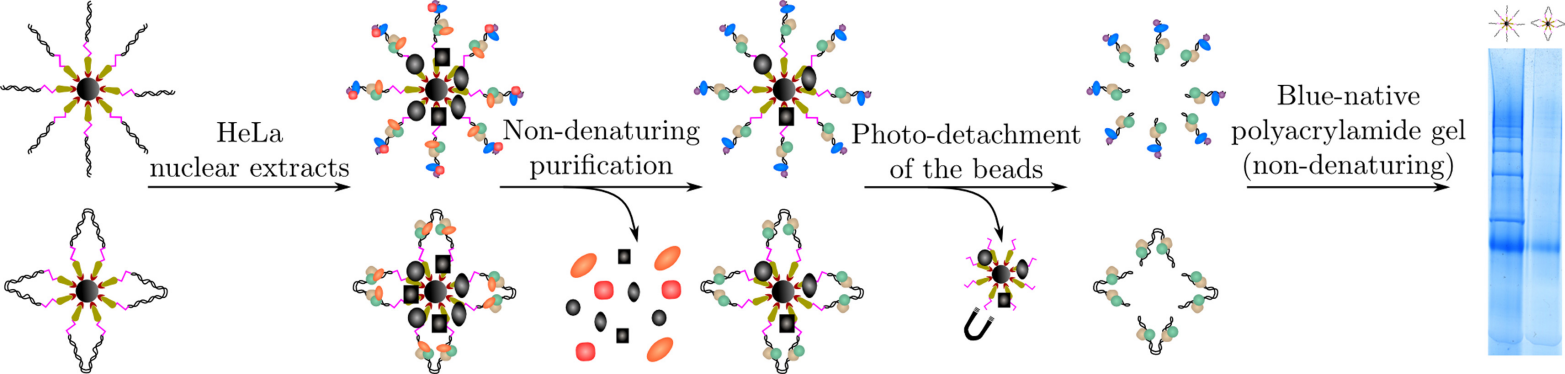
Two chromatographic phases were prepared and used to identify specific DNA ends-binding proteins. The upper chromatographic phase beared DNA ends, while the lower phase was devoid of such ends. HeLa nuclear extracts were used for the purification. After washing the chromatographic slurry, the purified nucleoproteic complexes were detached from the beads using UV illumination of the slurry. The purified material was subjected to a native-conditions polyacrylamide gel electrophoresis that showed very distinct migration patterns for the products obtained on each chromatographic phase. Each band was excised and its contents were identified by LC–tandem mass spectrometry following in-gel trypsin digestion.

The use of both the DNA ends phase and the control phase is at the basis of our analytical workflow (Figure 1). These phases are used to perform affinity purifications of DNA-binding proteins from HeLa nuclear extracts (see Materials and Methods) with the least stringent conditions possible for the washing steps, thanks to the detachment of the oligonucleotides from the beads (21). In order to monitor the diversity of the protein complexes assembled onto the chromatographic phases, we subjected the purification products to a blue native polyacrylamide gel electrophoresis (BN-PAGE (22,23)).

**Figure 2.**
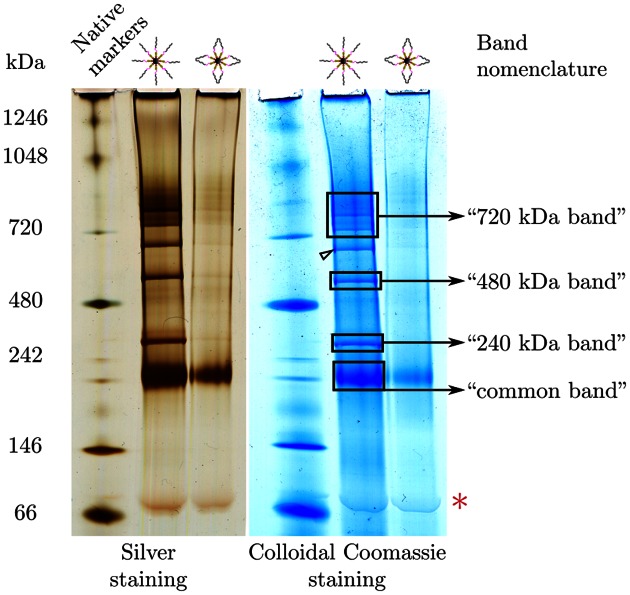
Blue native polyacrylamide gel electrophoresis of the purified samples. The nucleoproteic complexes purified either on the DNA ends phase or on the control phase were subjected to a BN-PAGE. The gel was cut into two halves that were either silver-stained or colloidal Coomassie blue-stained. The band nomenclature defines the band names as used later. The arrow head points to a band not systematically found in all experiments. The red asterisk indicates the migration position of almost naked DNA.

Figure 2 shows the typical results obtained after either silver or colloidal Coomassie blue staining of the native gel. Samples purified on the control phase, resolved into only one major band at an apparent mass of around 230 kDa. Samples purified on the phase exposing DNA ends, resolved into an unexpectedly clean band pattern with bands having apparent masses ranging from around 230 kDa up to more than 950 kDa. The band found at 230 kDa appeared to be common to both purification products, which is why we will refer to it later as the ‘common band’.

### DNA ends specificity of the nucleoproteic complexes

The purification of a number of protein complexes assembled specifically on the chromatographic phase exposing DNA ends strongly suggested that these assemblies had formed onto the very ends of the oligonucleotides. We thus asked if the BN-PAGE-resolved protein complexes still contained DNA after their migration in the gel. Using a radiolabeled oligonucleotide (Supplementary Figure S4, panel A), we observed that all of the Coomassie bands but the common one were mirrored on the autoradiography (panel B, ‘−’ lanes). This result indicated that the purified protein–DNA complexes did retain their DNA after their electrophoretic migration. The lack of radiolabel signal for the common band might be due to loss of either DNA or the label itself, as discussed later.

We then took advantage of the radiolabel at the distal end of the oligonucleotides to try to define further the location at which the purified complexes did assemble. For this, we subjected the purified nucleoproteic material to a thorough nuclease treatment with a cocktail of two highly active enzymes (see Materials and Methods). Upon BN-PAGE migration of the resulting complexes, the Coomassie staining of the gel showed that all the assemblies but the ones in the common band had a sizeable downward migration shift. The shifted bands still contained radiolabeled DNA, as demonstrated by the autoradiography (panel B, ‘+’ lanes). To determine if the protein complexes forming onto the DNA ends phase actually assembled at the very end of the oligonucleotide, the DNA contained in the various native gel bands (framed and numbered on the gel figure) was extracted and analyzed. Panel C shows a DNA sequencing gel in which the extracted DNA was migrated. After the nuclease treatment, the DNA molecules contained in the nucleoproteic complexes were all found to be ∼35 bp long. This result indicates that all the protein complexes, irrespective of their apparent mass, did assemble onto the very end of the oligonucleotides. This observation is compatible with the 16 bp minimal length of duplex DNA required to assemble protein complexes (29,30).

Altogether, these results show that the blue native PAGE separation of the purified nucleoproteic complexes preserves the association between the protein assemblies and the DNA oligonucleotides onto which they assembled. Over the set of six independent experiments on the DNA ends phase in this study, four major bands were reproducibly resolved by native gel electrophoresis for the purification products obtained on the DNA ends phase (Figure 2). These bands were systematically excised in pairs with the corresponding band in the control phase lane, even if not visible (see Supplementary Information for details on the sample pairing). Each pair of electrophoresis bands was then subjected to trypsin proteolysis and the extracted peptides were sequenced by LC–MS/MS to identify the proteins contained in the bands. Each protein identification could be associated to its semi-quantitation using the corresponding emPAI value (31,32). All the identification/semi-quantitation data have been injected into a database set up specially for this project and a graphical user interface program was developed to mine these data. Both the database and the data-mining program are freely available (see subsection Mass Spectrometry in the Supplementary Information section Materials and Methods).

**Figure 3.**
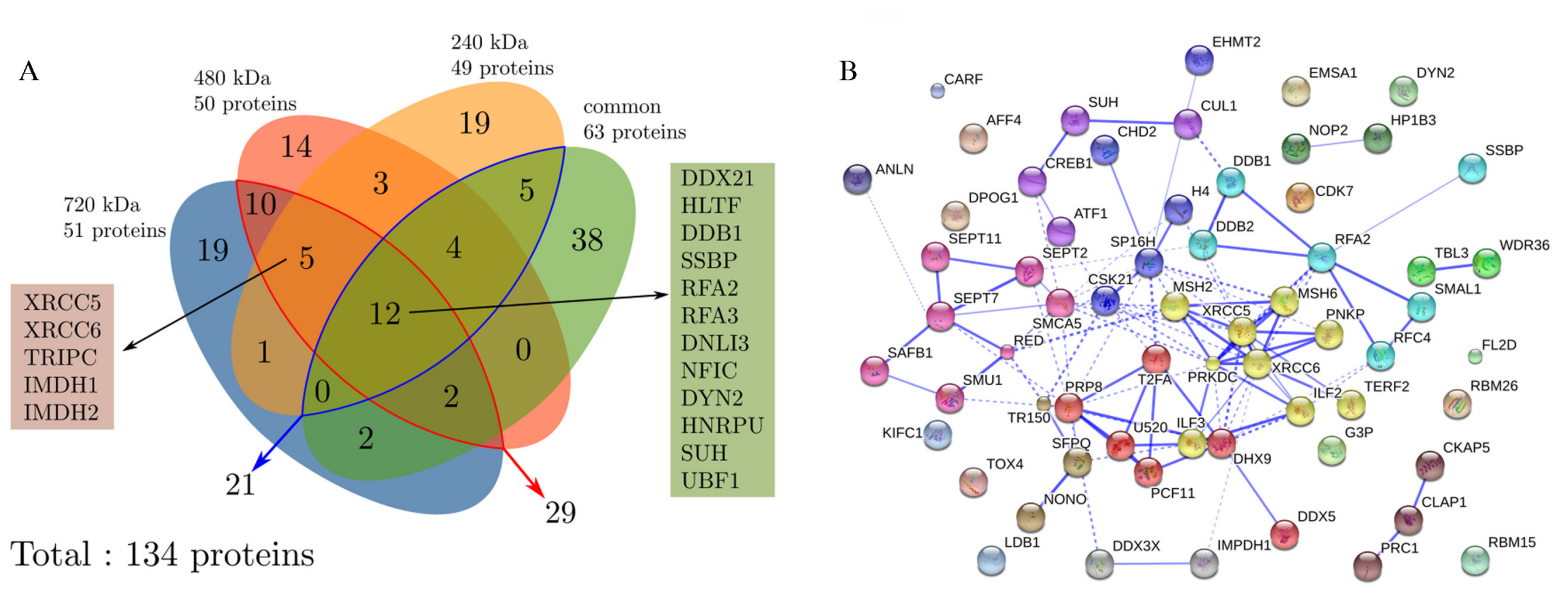
(**A**) Distribution of the proteins identified in the various bands after purification with the phase exposing DNA ends. Core proteome: the proteins common to all bands are shown in the green box, those present in all the bands but the common one are shown in the brown box. The proteins accounted for in this diagram were identified at least 4 times over the 6 independent experiments (see text). (**B**) Mapping of the proteins purified exclusively on the DNA ends phase on known interactomes using the STRING-8 framework (33). Clustering was performed based on the functional classes of the proteins, as represented by distinct colors. The lines show interactions between proteins that belong to the same cluster (continuous) or that belong to different clusters (dashed).

We assessed the quality of the separation of the different nucleoproteic complexes in the four distinct native gel bands by counting all the proteins that belonged to one or more bands. The Venn diagram shown in Figure 3A sums up the mass spectrometric identification results obtained for the different bands. One significant observation is that 90 proteins over the 134 identified at least 4 times in the 6 experiments were found to be exclusively purified in a given band. Another interesting observation is that among the 44 proteins that were shared across two or more bands, only 12 were actually shared by all the bands; this protein count reduced to only 5 when removing the proteins also found in the common band. Specifically, 29 proteins were found to be shared by the fully resolved 720 kDa and 480 kDa bands while only 21 proteins were shared by the two closely-migrating 240 kDa and common bands (see Figure 3A and gel in Figure 2). Overall, these data not only confirm our previous observations that the bands in these BN-PAGE gels contain supramolecular protein assemblies, but they also hint at a conspicuous polymorphism of the supramolecular complexes. These observations also attest the ability of the BN-PAGE to resolve nucleoproteic assemblies without any marked denaturation, since the majority of the proteins were found in one band only, as confirmed by the clean migration pattern, which did not suffer from any visible streaking. The proteins that were discriminated to be exclusively specific of the DNA ends phase (see below for the discrimination method) were mapped on known interactomes (33) (Figure 3B). This mapping provided an enlightening view of a number of interacting physiological pathways. Indeed, for example, proteins belonging to the cytoskeleton functional category, septins and anillin, were clustered together and had connections with another cluster, related to DNA repair. Conversely, we found that some proteins were not known to be involved in any interactome, like DYN2, which we consistently purified on the DNA ends phase.

### High DNA repair activity of the nucleoproteic complexes

As shown above, some of the complexes specifically assembled onto the DNA ends phase were of a high apparent molecular mass, suggesting that they might comprise the full set of double strand break repair proteins required to make them functional. To test the activity of the purified complexes, we devised a ligation experiment involving either the crude nuclear extract used for the purifications, as a control, or the purified complexes, in two different settings (see Materials and Methods).

**Figure 4.**
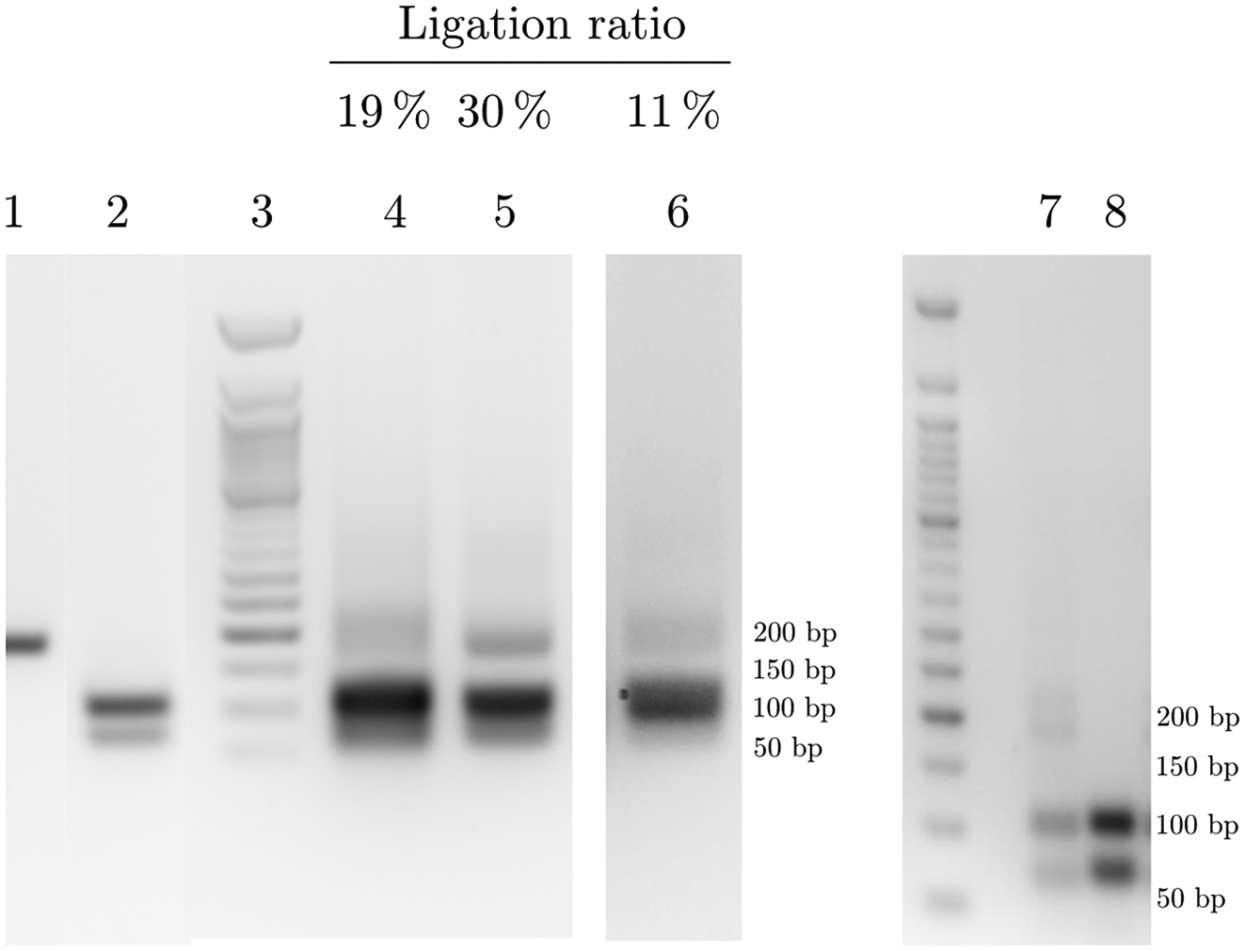
A ligation assay evidences that the purified nucleoproteic complexes are endowed with a DNA repair activity. (1) Control, full-length 186 bp DNA prior to Pst I cleavage; (2) Control, 101 bp and 78 bp fragments obtained after Pst I restriction of the full-length DNA, used to functionalize the beads; (3) 50 bp markers (DNA sizes on the right); (4) ligation products with the crude nuclear extracts; (5–6) ligation products with the purified nucleoproteic complexes either (5) after detachment from the beads or (6) prior detachment from the beads. A replicate experiment of lane (5) was performed with (8) or without (7) prior heating of the nuclear extracts. Lanes 1–6 were from a single gel, however the total amount of DNA recovered for lane 6 was reproducibly lower than for the other lanes and required increasing the signal/noise ratio by a factor 4. The ligation ratios are shown on top of the lanes.

Figure 4 shows the results of a representative experiment: the ligation ratio for the crude nuclear extracts (lane 4) was of 19 % and that ratio increased to 30 % for the purified nucleoproteic complexes after their detachment from the beads (lane 5). If the purified complexes were kept attached to the beads (lane 6), the ligation ratio was of only 11%. Heating the nuclear extracts prior to the ligation step abolished the ligation, showing that the extracts contained the ligating activities. These results are interesting because they show that the DNA repair complexes purified on our DNA ends phase are functional and also highlight the benefit of our purification strategy, since the detachment of the purified nucleoproteic complexes from the chromatographic support prior to the ligation led to a significant increase in the repair activity. The observation that the ligation activity is less efficient when the nucleoproteic complexes are still attached to the beads is easily explained by the fact that the beads, even upon gentle agitation of the sample tube during the ligation, tend to limit the diffusion freedom of the nucleoproteic complexes, which limits the successful ligation reaction. Another noteworthy observation is the fact that the ligation assay performed with the crude nuclear extracts involved a total protein amount of 320 μg while the assay performed with the purified nucleoproteic complexes involved protein amounts in the range 5–10 μg. Nonetheless, the ligation ratio was significantly higher in the latter case, showing that the specific enrichment of the DNA repair complexes onto our chromatographic phase is very high and that these complexes are highly functional.

### The DNA ends proteome

**Figure 5.**
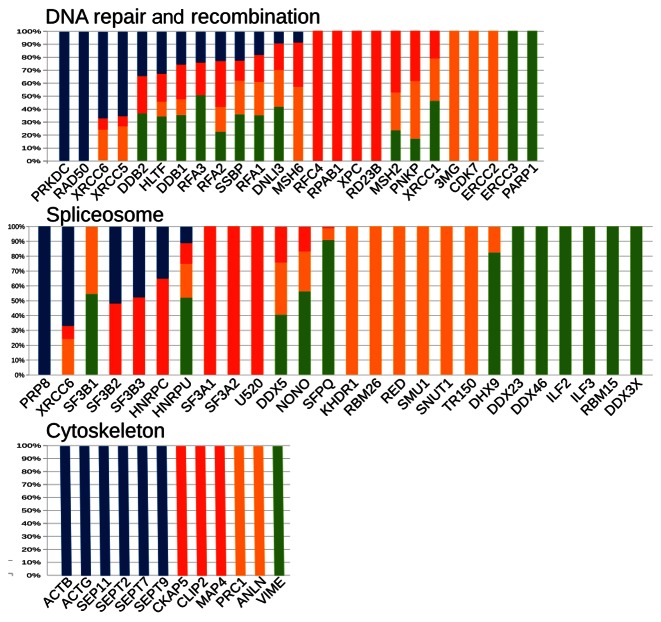
Examples of functional classes showing proteins differentially distributed into one or more bands. The proteins accounted for in this diagram were identified at least 3 times over the 6 experiments. Band color code (see Figure 3A): blue, 720 kDa; red, 480 kDa; yellow, 240 kDa; green: common). See Supplementary Table S8 where all the proteins specific of the DNA ends phase are listed along with their relative distribution in the various bands.

Gathering all the protein identifications obtained for the various bands resolved starting with the material purified on the DNA ends phase and subjecting them to the KEGG Brite pathway analysis and ontology software permitted their classification into a set of functional classes (note that a protein might belong to more than one such class (34)). The set of histograms in Figure 5, S5, S6 and S7 shows, for each functional class, how the various proteins—purified using the DNA ends phase—distributed in each of the four bands.

Figure 5 specifically addresses three exemplary cases: DNA repair and recombination, spliceosome and cytoskeleton. Roughly half of the proteins in the DNA repair and recombination class were found to be shared across more than one band. For example, XRCC5 (Ku80) and XRCC6 (Ku70) were found to be shared among all bands but the common one; the DNA ligase 3 (DNLI3) and SSBP were shared among all the bands. The other half of the proteins were identified in only one of the four bands (e.g. PRKDC (DNA-PKcs) was only found in the 720 kDa band, RD23B and XPC only in the 480 kDa band, CDK7 only in the 240 kDa band and finally PARP1 only in the common band). For the spliceosome and cytoskeleton functional classes, the proteins were either much less (spliceosome) or not at all (cytoskeleton) distributed among multiple bands. These observations highlight a markedly greater propensity of DNA repair and recombination proteins to assemble into diverse complexes when compared to the cytoskeletal or spliceosomal ones.

The chromatographic phase exposing DNA ends used for the experiments described above necessarily contained the internal sequence of the duplex oligonucleotide. We therefore asked which of the proteins purified with that phase did actually reflect the DNA ends proteome. In order to filter out the proteins interacting with the internal sequence of the DNA oligonucleotide functionalizing the beads, we used the proteins purified during the matching control experiment, on the control phase not exposing DNA ends, to define an index estimating the specificity of the proteins for the DNA ends (see Materials and Methods). In the Supplementary Figures S5–S7, the bottom histograms present the same data as in the top ones but after having applied the following filter: all proteins having *I* < 0.9 were removed. That filter was highly efficient in the removal of a conspicuous number of proteins not showing a great specificity for the DNA ends (Supplementary Tables S8 and S9 list proteins exclusively specific of the DNA ends phase, with *I* ≥ 0.9). Indeed, at least 70% of the proteins belonging to the Chromosome and the DNA replication classes were removed. Interestingly, only 56% of the proteins in the DNA repair and recombination class were filtered out, suggesting that they had a stronger intrinsic specificity for the DNA ends. It is worthwhile to note that in any of the various functional classes, at least 25% of the identified proteins were retained after the filtering. Altogether, these observations suggest that the chromatographic strategy used in this report is particularly appropriate to define a highly specific DNA ends proteome. As shown in Supplementary Figure S5, considering the bottom histogram of the DNA repair and recombination functional class, three major proteins of the double-strand break repair pathways—XRCC6, XRCC5, PRKDC—were retained after the filtering. Conversely, the proteins XRCC1 and DNLI3, known to be involved in single-strand break repair, were filtered out. Other proteins were unexpectedly found to be retained upon filtering: CDK7, although it is known to be indirectly part of the DNA damage response and DDB1/2, involved in the nucleotide excision repair. Supplementary Figure S6 shows the filtering effects for the proteins involved in the spliceosome functional pathway. Most interestingly, the NONO and SFPQ proteins, that were initially discovered as spliceosome-specific proteins, were retained upon filtering, which is consistent with a more recently-discovered involvement in the non-homologous end-joining pathway (35,36). In the same way, proteins ILF2 and ILF3 were retained, which is consistent with their connection to the non-homologous end-joining pathway (37). Conversely, splicing factor subunits SF3A1/2 and SF3B1/2/3 were removed. Supplementary Figure S5 shows the data pertaining to the proteins of the DNA replication functional class, where we observe the interesting case of the telomeric repeat-binding factor 2 (TRF2/TERF2). Indeed, TERF2, which is known to interact with either XRCC6 or the KU dimer (38–40) is retained after filtering and is present in the same three bands as the KU dimer. Conversely, the topoisomerases TOP2A, TOP2B and TOP1 are removed by our filtering process, as expected, because these proteins are known to be removed from the telomeric DNA by TERF2 (41).

### The DNA repair subproteome on the DNA ends

**Figure 6.**
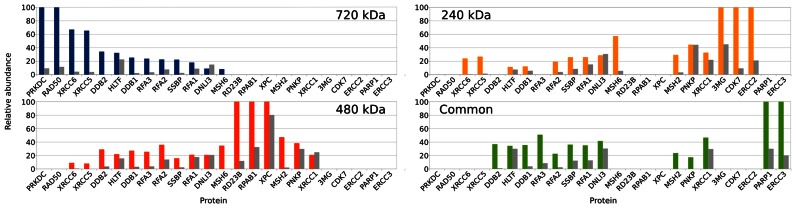
Distribution of the proteins identified in the various bands after purification with either the control phase (grey bars) or the DNA ends phase (colored bars). The proteins accounted for in this diagram were identified at least 3 times over the 6 experiments (see text). Proteins were sorted by their relative abundance in the 720, 480, 240 kDa and common bands, in that order. See Supplementary Table S9 where all the proteins specific of the DNA ends phase are listed along with their DNA ends phase specificity index reported for each band.

Overall, the previous results showed that the proteins involved in DNA repair and recombination were the most consistently distributed in the set of four native gel electrophoresis bands, as viewed in the corresponding histogram, where the bars were the most consistently multicolored. Indeed, over the 44 proteins shared among two or more bands, 14 proteins are directly related to DNA repair (see Figure 3A). Further, from a quantitative standpoint, four of the five most abundant proteins purified on the DNA ends phase are involved in DNA repair, like DNA-PKcs, Ku70, Ku80 and PARP1. This was expected, because the chromatographic phase exposing DNA ends mimicked double-strand breaks and it is well known that such ends are able to recruit DNA repair complexes *in vitro* (42,43). Consistently, the most abundant DNA repair proteins belong to the non-homologous end-joining pathways. Supplementary Figure S5 showed that the DNA repair proteins divided in all the four bands of the native gel, with different proportions in each of these bands and that this class of proteins had a relatively low filtering rate, indicating that these proteins had highly specifically bound to the DNA ends phase. Figure 6 shows, for each of the four bands, the relative amount of each protein as purified either on the control phase or on the DNA ends phase (see Materials and Methods) and Supplementary Table S8 lists all the proteins found to be specific of the DNA ends phase along with their proportions in the various bands.

A set of proteins known to be directly involved in the double-strand break repair pathways were identified as highly specific of the DNA ends phase, like the proteins DNA-PKcs, XRCC5 and XRCC6. As was expected, a number of proteins of the various DNA repair pathways were found to be unspecific of the DNA ends. For example, the DNA ligase III, XRCC1, XPC and HLTF were systematically found to be equally recruited on both phases. PNKP is an interesting example on two grounds. First, it shows a varying specificity for the DNA ends depending on the complexes to which it associates: it is found to be highly specific for the DNA ends phase when interacting with protein complexes in the common band but not for complexes in the 240 kDa or in the 480 kDa band. Second, it shows up in bands when other proteins are also found, as is the case for XRCC1 with which it is well known to interact directly (44,45). As an interesting sidenote, we observe that PNKP is absent from complexes that migrate in the 720 kDa band, that in turn comprise DNA-PKcs. It is still debated whether the catalytic subunit of the DNA-dependent protein kinase is required to be present through all of the repair process. In a widely-accepted model, DNA-PKcs undergoes autophosphorylation, detaches itself from the DNA ends thus allowing new proteins to assemble on the ends (46). More recent works tell a different story: the NHEJ DNA repair complexes assemble dynamically not necessarily in an ordered and sequential manner (47,48). In our experiments, PNKP is found assembled onto the DNA ends exclusively in the absence of DNA-PKcs.

XRCC5 and XRCC6 are found in the complexes that migrate in all the bands except the common one. We observed earlier that this latter band did contain much lower amounts of intact DNA as shown by the absence of the radiolabel in the results described in Supplementary Figure S4. The removal of the radiolabel might have been due to nucleolytic activities acting on the oligonucleotide's distal end. The absence of KU from the common band might have left the DNA ends unprotected, thus favoring the access to other damage-sensing proteins of lower end-protecting efficiency than KU, like PARP1 and SSBP.

MSH2 and MSH6 are collectively documented to be part of more than 26 different complexes (as reported by Corum (49)) of which around 20 deal with the mismatch repair pathway (MMR) and the remaining ones with other functional pathways; specifically, they contribute to the genome stability (50). These proteins were systematically purified only on the DNA ends phase, which is consistent with their known interactions with KU and DNA-PKcs and with the role of MSH6 in the activation of NHEJ (51). Strikingly, their distribution in the different bands perfectly matches their interactome in the literature (51): MSH6 interacts with DNA-PKcs while MSH2 does not, as reported by Biogrid (52), consistent with our observation that MSH6 is purified in the 720 kDa band, while MSH2 is not.

The DDB1 and DDB2 proteins are systematically purified on the DNA ends phase. This is a rather unexpected result because these two proteins are involved in the UV damage DNA repair (NER) as is XPC, with which they copurify (53). The fact that XPC is essential for NER but not specific of the DNA ends phase suggests that the recruitment of the DDB proteins onto the DNA ends is independent of NER. Further, DDB1 and DDB2 also copurify with other known interactors, like RPA (54).

The cyclin-dependent kinase CDK7 is particularly interesting because, though it is known to be involved in the DNA damage response at a later stage than that of the detection of DNA damage, specifically at the level of cell cycle regulation, we find it purified on the DNA ends phase. This unexpected result might highlight a CDK7 role in an early stage of the DNA damage response.

Finally, a general outlook at Figure 6 is that the DNA ends exposed by our chromatographic phase are specifically dealt with by the canonical NHEJ pathway (mainly in the 720 kDa band) but unexpectedly also by the NER (mainly in the 480 kDa band), the MMR repair pathways (all bands) and the BER pathway (common band), this latter pathway sharing a number of proteins with the alternative NHEJ pathway. The interactions of all the identified proteins with any subunit of the DNA-dependent protein kinase are specified in the last column of Supplementary Tables S8 and S9.

### Unexpected members of the DNA ends proteome

A number of proteins specifically recruited onto our DNA ends phase have no known involvement in DNA ends-related processes. The most striking example is dynamin (DYN2) (55). This protein was consistenly present in 92% of all the material purified on the DNA ends phase, in all the bands, and was systematically absent from the control phase-purified material. The fact that DYN2 is systematically present in all of the four bands suggests that it participates to a number of different complexes. These observations argue in favor of a direct interaction of DYN2 with DNA, which is intriguing because this protein is a molecular motor involved in cytoskeleton dynamics and cytokinesis (56,57). In an analogous manner, septins (58,59) and the anillin protein (ANLN) (60) are also involved in actin-based cytoskeleton physiology and cytokinesis, without any known physical interaction with DNA. The cAMP response element binding protein 1 (CREB1) is a transcription factor that controls a huge number of genes. This factor has been described as a target of the ATM kinase in response to genotoxic stress (61), without having ever been shown to be directly involved in the DNA ends proteome and even less in the DNA damage response. Interestingly, ATF1, which is structurally related to CREB *via* its carboxy-terminal basic region/leucine zipper motif (62) was also purified in the same band as CREB1 (see Supplementary Figure S7). The involvement of TR150, that is purified in the 240 kDa band, in the specific binding of DNA ends is not well documented, although a study shows its participation in the DNA damage response. TR150 (TRAP150 or THRAP3), a protein involved in the splicing process, has also been shown to be actively involved in DNA repair because its depletion produces a hypersensitivity to DNA damaging agents (63). We found that the well-known housekeeping genes G3P (GAPDH) and IMDH1 are specific of the DNA ends phase. These proteins are not documented for their interaction with DNA ends. In the case of IMDH1, its recruitment on our DNA ends phase is reminiscent of the ribonucleotide reductase (RIR2B) that is known to supply deoxyribonucleotides for DNA repair in cells arrested at cell cycle phases G1 or G2 (64,65). The NOP2 (66), DDX3X (67) and PCF11 (68) proteins are involved in the RNA physiology and are another example of how DNA and RNA physiological processes are intertwined (see Discussion). The H4 histone (69) was astonishingly purified in a distinct manner with respect to all the other histone proteins. Indeed, it purified in both the 720 kDa and 240 kDa bands and was never found in the 480 kDa band. Conversely, all the other histone proteins were purified in the common band. These results are intriguing because all these proteins could have been expected to be purified also on the control phase. Proteins RED, RBM26 and SMU1, which are involved in the spliceosome, were purified in the 240 kDa band exclusively while WDR36 and TBL3, which are involved in the ribosome biogenesis, were purified exclusively in the 480 kDa band.

The histogram in Supplementary Figure S7 shows the proteins that were purified exclusively on the DNA ends phase and that were unclassified by the KEGG Brite software. Indeed, these proteins are almost not documented in the literature. Strikingly, of all nine proteins, eight are only purified in one single band, with the remaining one (SUH) being found in all four bands. One of these proteins is CARF (collaborator of ARF) that was shown to be involved in the DNA damage and checkpoint response of cells through ATM/CHK1/CHK2, p53 and ERK pathways (70). Similarly, SUH and SP16H are also involved in the DNA damage response, either in the Notch signalling pathway or as a constituent of the FACT complex, respectively (69,71). The scaffold attachment factor SAFB1 contributes to the DNA damage-induced spread of the γH2AX response signal by allowing the chromatin remodeling around the DNA damage site (72). It is well known that the γH2AX response signal originates at the DNA damage and propagates to very long distances. The fact that SAFB1 acts at an early stage of the DNA damage response, with its location being precisely at the DNA damage, might explain why we specifically purify it on the DNA ends phase. The proteins FL2D (73) and HP1B3 (74) are involved in the regulation of the cell cycle progression. To the best of our knowledge, the three proteins EMSA1, LDB1 and TOX4 have no known function.

## DISCUSSION

The presence of DNA ends is critical to the cell, which deals with them using a large set of proteins belonging to various physiological pathways like DNA repair, telomeric end protection or variable domain junction (VDJ). It had long been thought that DNA ends were recognized only by a definite set of proteins specifically involved in DNA repair or DNA ends protection. We report here an unexpected diversity of proteins belonging to various physiological pathways that were specifically purified on a chromatographic phase exposing DNA ends. We thus termed the whole set of such proteins the DNA ends proteome. One remarkable observation that kept repeating itself throughout this work is that there is an astonishingly high level of consistency between the composition of the purified complexes resolved by BN-PAGE and the known functions of the molecular partners of these complexes (see the examples of TERF2, NONO and SFPQ, PNKP and MSH2/6 earlier in this report). This observation reinforces our confidence that the unexpected proteins that we found consistently purified on the free ends phase are indeed part of these complexes and open a path to new research on the ways DNA ends are dealt with by cells (see the peculiar examples of cytoskeleton-related proteins like DYN2 (dynamin), ANLN (anillin) and septins).

From a technical standpoint, the immediate visualization of the purified protein–DNA complexes allowed us to focus our attention on four very well-resolved bands and thus to limit our analyses to selected complexes of known apparent molecular masses: all the proteins not involved in non covalent protein assemblies formed on our DNA phases during the purification process were dropped. The proteic content of the various native gel-resolved bands reflected the presence of complexes differing greatly one from another, strongly suggesting that complexes of lower apparent molecular mass did not form as a result of the denaturation of heavier ones.

The purified proteins were systematically identified by tandem mass spectrometry using stringent parameters, specifically the requirement to have at least three sequenced peptide matches to successfully identify a protein, unlike the general high-throughput pipelines that require only two matches. The data set described in this report was obtained from six independent experiments. The data published in this report pertain only to the proteins that were identified in at least three experiments over the six total experiments that were performed. The semi-quantification of the proteins was based on a label-free technique involving the calculation of the emPAI value of each protein. Using the emPAI value of different proteins to compare their abundance is inherently unreliable, because the differing protein sequences might produce peptides of differing ionization abilities during the mass spectrometric analysis. We therefore only compared emPAI values relative to a same protein in different bands and/or samples in all the data-mining procedures described in this report. Two main emPAI-based semi-quantitation comparisons were performed throughout this work. First, we computed for each purified protein a specificity index that measured its tendency to be specifically recruited on the DNA ends phase versus the control phase. Upon removal of all the proteins with a specificity index less than 90%, we showed that this emPAI-based comparison method is powerful, because it highlighted proteins well known to be specific for the DNA ends, like PRKDC (DNA-PKcs), Ku70 (XRCC6), Ku80 (XRCC5). Further, it allowed to single out a number of proteins that were not yet known to be directly involved in DNA ends-related processes, like the proteins DDB, PRP8, CDK7, SP16H and FL2D, or proteins clearly involved in the interconnection of various functional categories (DHX9, TR150, RFA2, SMCA5). Second, we performed abundance comparisons of proteins found in two or more bands of the same purification product, i.e. bands from the same gel lane. This kind of comparison proved highly useful and reliable because it showed, for example, that the Ku70:Ku80 dimer was much more abundant in the 720 kDa band, where the catalytic subunit of the DNA-dependent kinase (PRKDC) was also found, than in the 480 kDa band, where the *Xeroderma pigmentosum* group C-complementing protein (XPC) was found, which is known to be antagonized by KU in the context of UV damage (75). Further, these quantitations showed that the KU protein was absent from the common band, while PARP1 was found only in this band, which is consistent with reported results (76). All these results demonstrate that the nucleoproteic complexes resolving in the gel bands faithfully reflected the composition of biological assemblies that perform, depending on the cellular context, different tasks.

Large-scale protein–DNA interactions studies have always faced the overwhelming abundance of determinate classes of proteins. For example, endeavors to characterize the transcription factors interactome of a given DNA sequence are hampered by the massive recruitment of DNA repair proteins on the bait DNA duplex oligonucleotide (20,77). In an interesting twist to these observations, the proteome that we report here encompassed a large set of protein functional classes of which the DNA repair proteins represented only 17% (although some additional proteins should have been classified by the KEGG Brite software in this class). The remaining proteins belonged to 11 well-documented protein classes and another class of proteins of unknown function. As detailed above, two sets of DNA ends-specific proteins are particularly intriguing: the proteins of undocumented function and the proteins that were unexpectedly purified on the DNA ends (dynamin and septins, for example). Overall, these results highlight the ability of the methodologies described in this report to afford a highly specific and unbiased proteome of the free DNA ends.

In an attempt to define a core DNA ends interactome, the proteins found to be simultaneously localized in either the four electrophoretical bands or only the three heaviest ones, have been listed in Figure 3A. Although not numerous, these proteins do belong to as much as seven different functional classes. Some of these proteins are known to be part of a great number of different complexes, which is consistent with our observation that they purify in various bands of significantly differing apparent molecular masses.

The fact that the most abundant proteins recruited specifically on our DNA ends chromatographic phase were DNA repair proteins suggests that the assembly of the protein–DNA complexes resolved by native gel electrophoresis is driven by DNA repair proteins, like KU, which is remarkably abundant in the nucleus. Indeed, it is noteworthy that the differential purification of nucleoproteic complexes on both the DNA ends phase and the control phase is mostly visible on the blue native gels for bands that do contain the KU dimer, i.e. all the bands but the common one.

The KU dimer and the catalytic subunit PRKDC of the DNA-dependent protein kinase are known to be involved in an astounding number of protein complexes (78). One interesting observation, in this respect, is that only 20% of the DNA ends proteome reported here has known interactions with any of these three proteins (using the Biogrid engine (52)), which suggests that a conspicuous part of the remaining proteins display a DNA ends phase specificity unrelated to DNA-PK (e.g. DDB1). Indeed, a number of DNA repair proteins were specifically recruited onto our DNA ends phase even when they belonged to DNA repair pathways not involved in the repair of double strand breaks (MSH2, MSH6, DDB2), which might evidence an early cross-talk of different DNA repair pathways occurring on our chromatographic phase (75). These observations strongly suggest that our methodology is indeed able to single out proteins interacting with partners in the nucleoproteic assemblies in manners not yet documented.

One interesting observation is that the DNA ends proteome is heavily populated with proteins mainly documented in the literature for their involvement in RNA physiology. There is, however, an increasingly documented cross-representation of the RNA-specific proteins in DNA-based screens (79). In our DNA ends proteome, known RNA-specific proteins were found to belong to various functional classes, like the spliceosome or the RNA biogenesis classes. The spliceosome class was particularly well populated, with a number of proteins having mixed functions in the DNA and RNA realms. XRCC6 is mainly known as a DNA repair protein; its involvement in RNA splicing has been only rather recently uncovered (80). Conversely, SFPQ and NONO have been discovered as RNA splicing proteins that only recently were assigned DNA repair functions (35,36,81,82).

To conclude, the fundamental basis of this work was to elaborate a new methodology allowing the purification of DNA ends-specific proteins with no preconception whatsoever about the proteins to be searched for. Indeed, our naive purification approach did show that there are still protein–protein interactions awaiting discovery in the field of DNA repair, specifically involving proteins not known to be part of any of the DNA physiology-related functional classes. As such, this work sets the stage for straighforward investigations of DNA ends interactomes in various cellular genetic backgrounds, like cells mutated or deleted for specific proteins of interest. Also, it may be used for the exploration of the physiological effects of DNA ends-producing agents, like ionizing radiations or other genotoxic agents. Further, specific chromatographic phases can be prepared to incorporate modified DNA oligonucleotides, bearing clustered DNA damages or complex chemistry ends, like those produced by ionizing radiations. The unraveling of a highly connected network of proteins belonging to an unexpectedly wide functional spectrum promotes the idea that cancer research should strengthen the diversification of its targets.

## Supplementary Material

Supplementary Data

SUPPLEMENTARY DATA
