# Supplementary material for: The human DNA ends proteome uncovers an unexpected entanglement of functional pathways
Source: Nucleic Acids Res. 2016 Feb 25;44(10):4721–33. doi: 10.1093/nar/gkw121 (PMC4889927; doi:10.1093/nar/gkw121)
Supplement: Supplementary Data [file gkw121_Supplementary_Data.zip › nar-02937-m-2015-File002.pdf]

The human DNA ends proteome uncovers an unexpected entanglement of functional pathways

Supplementary information

Vivien Berthelot, Gildas Mouta-Cardoso, Nadia Hégarat,
François Guillonneau, Jean-Christophe François,
Carine Giovannangeli, Danièle Praseuth and Filippo Rusconi

MATERIALS AND METHODS

Sampling scheme

For the data mining described in this work, the set of samples, as divided in two the two ✧ and ✳ categories, is shown below (sample numbers are categorized by band identity). The protein identification data obtained from all the listed samples populate that published database. Each ✧ sample is compared to the corresponding ✳ sample. For example, sample vb96 is the control sample of vb81. Note how the control sample is used twice, as a control experiment for vb81 and vb85 (two samples coming from a same-conditions comparable but not technical replicate experiments).

Band	✧	✳
720 kDa	vb96,vb96,vb111,vb119,vb129,vb134	vb81,vb85,vb104,vb112,vb120,vb130
480 kDa	vb97,vb97,vb110,vb118,vb127,vb135	vb82,vb86,vb105,vb113,vb122,vb131
240 kDa	vb98,vb98,vb109,vb117,vb126,vb136	vb83,vb87,vb106,vb114,vb123,vb132
common	vb99,vb99,vb103,vb108,vb116,vb137	vb84,vb88,vb107,vb115,vb124,vb133

All the data used in this work are available as spreadsheet files bundled together in the Supplementary information (file berthelot-et-al-spreadsheet-formatted-mass-data.zip). Each sample above has a corresponding spreadsheet file associated with it. The spreadsheet file contains tabs describing the protein identification data, along with each gas phase-sequenced peptide data. Altogether, these data represent a tabulated version of the X!Tandem-generated identification data and match precisely the data in the database made available on the internet (see below).

Mass spectrometry

The raw mass data were converted to XML-formatted data files using the msconvert program of the Proteowizard software suite running in MS-Windows. The output format was mzXML, with TPP compatibility enabled and 64-bits binary encoding precision. All the remaining data processing steps were performed on a Debian GNU/Linux platform (http://www.debian.org) using the following set of software programs: the X!Tandem protein identification software that uses tandem mass spectrometry data along with the SwissProt protein database restricted to the human proteins; the X!TandemPipeline software that interfaces with X!Tandem and provides useful features to both filter and group X!Tandem-generated protein identifications (http://pappso.inra.fr/bioinfo/xtandempipeline), the Sqlite3 database (http://www.sqlite.org) and home-made software. Two pieces of software were specifically developed in-house; the first C++ software was developed to parse the XML-formatted data generated by X!TandemPipeline and inject mass data into the Sqlite3 database, and the second C++ software—freeDnaEndsProteome—was developed to allow an easy data mining of the bio-structural data in that populated database. This software and its detailed user manual are made freely available, along with the database itself (see below).

The X!Tandem database searching program was configured with the following settings: maximum precursor ion charge: 3; fragment mass error: 0.5 Da; minimum ion count: 4; maximum missed cleavage sites: 1; b,y ions. A protein was identified only if at least two unique peptides matched its sequence.

The false discovery rate (FDR) assessment was performed by applying the target-decoy approach at the X!Tandem database search step. The spectra were thus searched twice by X!Tandem, once against the target database and once against a decoy database prepared by reverting the sequence of all the proteins. In our set of data, the FDR peaked at 0.6 %; overall, the samples did contain a maximum of a hundred identified proteins.

For the PAI calculation, only the theoretical tryptic peptides in the mass range 800–2500 Da were considered to be “observable”.

FIGURES AND TABLES

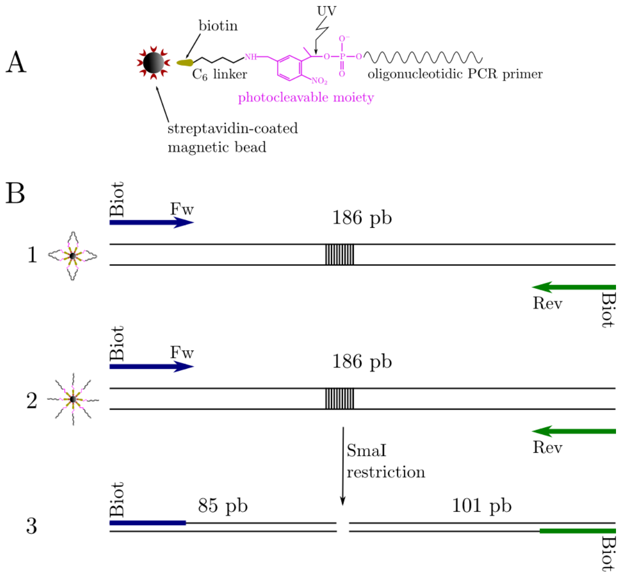

Fig. S1: Home-made affinity chromatography phases. (A) Structure of the photocleavable linker. (B) Scheme representing the production of bibiotinylated (1) and monobiotinylated (2) DNA duplexes. Restriction by Sma I produces two fragments of 85 and 101 bp (3). DNA oligonucleotides of sizes less than 186 bp (58 bp and 74 bp) were tested initially but did not yield a correct differential between the sets of proteins purified on the monobiotinylated phase *vs* those purified on the bibiotinylated phase.

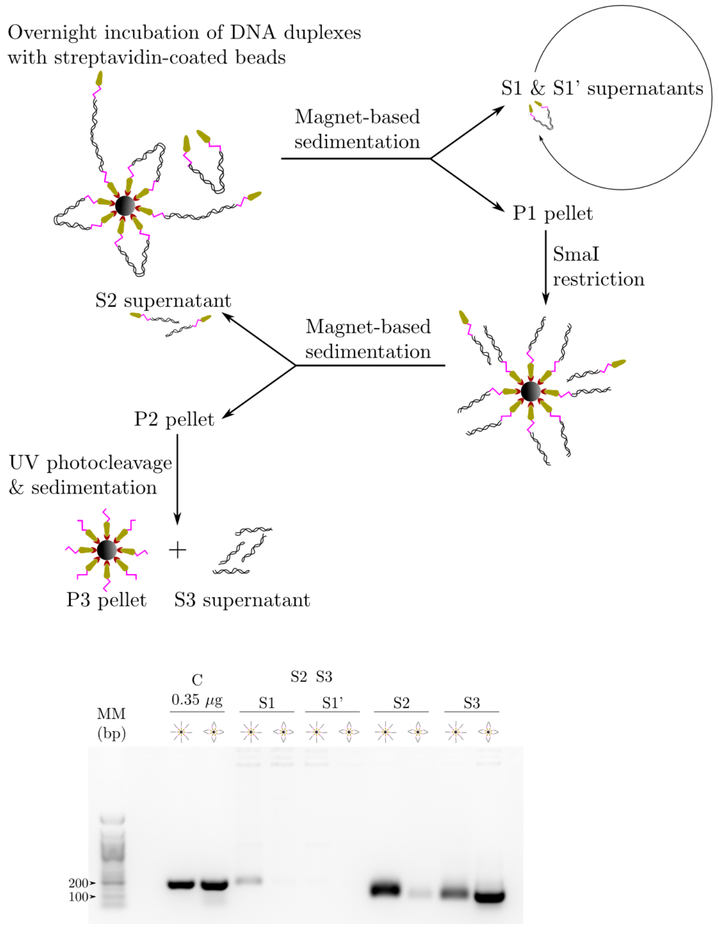

Fig. S2: Verification of the *bona fide* constitution of the chromatographic phases, either devoid of free DNA ends (control phase;) or endowed with such ends (DNA ends phase;) [the scheme only shows the process for the bibiotinylated DNA but is identical for the monobiotinylated DNA]. Biotinylated duplex DNA oligonucleotides are incubated with the streptavidin-coated beads. The beads are magnet-sedimented and the supernatant is recovered (S1). The beads are washed once more (S1'). The pelleted beads are then resuspended and treated with Sma I. Following the endonucleolytic digestion, the supernatant is collected (S2) and the beads are subjected to UV irradiation to detach the remaining DNA material, that is collected in the supernatant (S3). All of the collected supernatants are deposited onto an agarose gel. Lanes S1 and S1' of the agarose gel show that, following incubation of the biotinylated DNA duplex oligonucleotides with the beads, almost all of that material was effectively bound to them. The S2 supernatant contains a significant amount of material for the DNA ends phase ( ) ; a faint band for the control phase ( ) showed that the Sma I restriction of the bibiotinylated oligonucleotides did release a very low amount of DNA material. Following the photocleavage of the material still bound to the beads, the S3 supernatants were recovered and analysed. Lanes S3 show a thick band that contained two DNA species: 85- and 101-bp fragments, as expected following digestion of the oligonucleotides with Sma I. The band is more intense for the phase than for the phase because the amount of DNA released from is twofold the amount of the DNA released from . Overall, these results show that the bibiotinylated duplex DNA oligonucleotide did indeed loop onto itself, leading to the production of a chromatographic phase effectively devoid of free DNA ends ( ). C: 0.35 μg of undigested control DNA corresponding to a fifth of the DNA that was loaded onto the beads. MM: 50 bp molecular markers.

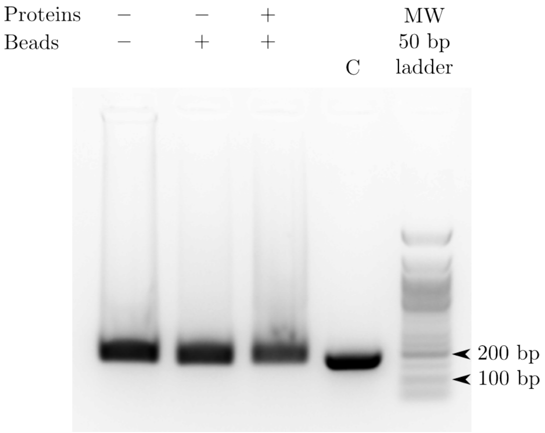

Fig. S3: Verification of the potential effects of UV irradiation on the DNA oligonucleotides. The monobiotinylated duplex DNA oligonucleotide was used to monitor the potential UV-induced DNA damage. The purification process was performed with the usual procedure on a monobiotinylated chromatographic phase (✳), either with ($^{+}_{+}$) or without ($^{-}_{+}$) the nuclear extract. An irradiated control sample was monobiotinylated DNA that had not undergone interaction with neither the beads nor the proteins ($^{-}_{-}$). Non-irradiated monobiotinylated DNA was deposited in C as a migration control.

Following the purification, the samples were irradiated, the beads were magnet-pelleted and the supernatant underwent the following steps (same treatment for the control sample that migrated in the ($^{-}_{-}$) lane above):

1. Protein digestion was performed by Proteinase K at 55°C for 30 min. The mixture was then phenol/chlorophorm/isoamyl alcohol-extracted and ethanol-precipitated;

2. Treatment with the DNA damage-specific nuclease that nicks at the position of cyclobutane pyrimidine dimers was performed using the T4 PDG endonuclease according to the manufacturer's instructions (New England Biolabs);

3. Treatement with the T7 endonuclease to convert nicks to double-strand breaks was performed by bringing the sample to 10 mM Tris-HCl and 10 mM $MgCl_2$ (pH 8.5);

4. A second protein digestion (Proteinase K) and phenol/chlorophorm/isoamyl alcohol extraction were performed, followed by ethanol precipitation;

5. Gel electrophoresis was done on a 1.8 % agarose gel.

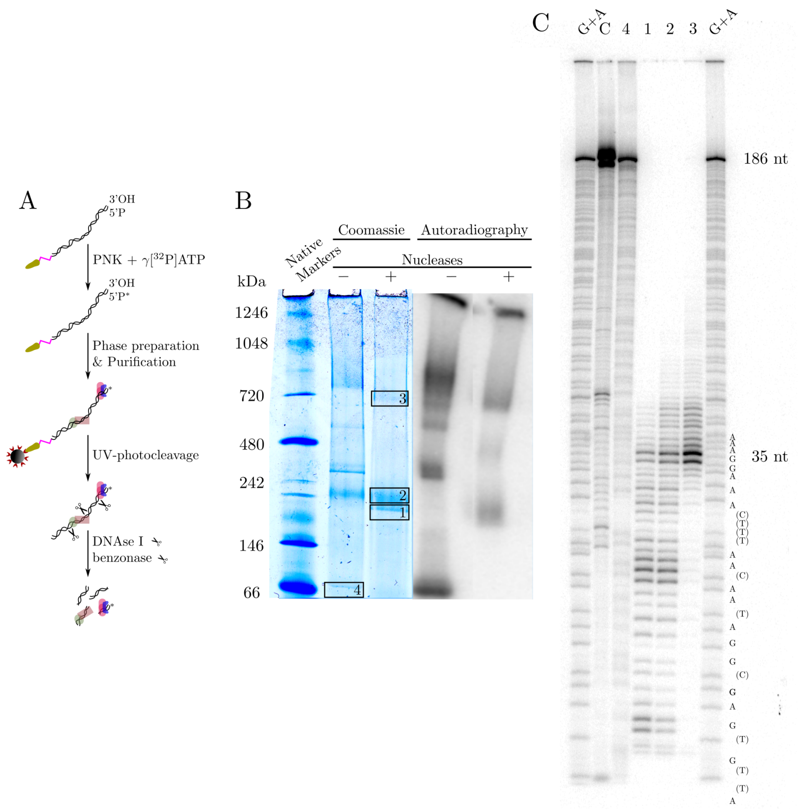

Fig. S4: The protein complexes did assemble onto the duplex DNA oligonucleotides functionalising the beads. (A) The monobiotinylated oligonucleotides used to prepare the chromatographic phase are radio-labelled. The phase preparation and the purification are then carried over normally. The purified nucleoproteic complexes are digested using DNAse I and benzonase. (B) The purified complexes are subjected to a BN-PAGE and the gel is then autoradiographed. Note how bands in the untreated lane (-) are shifted downwards in the treated lane (+). (C) Bands from the gel (numbers) are excised and the contained DNA is extracted and subjected to a DNA sequencing electrophoresis. Lanes G+A contain the DNA fragments obtained by Maxam and Gilbert sequencing of the DNA oligonucleotide produced by PCR and lane C contains that same DNA oligonucleotide but unmodified, as a migration reference for an intact 186 nt DNA. Lanes 1–4 contain the DNA extracted from the corresponding bands in the gel in panel B.

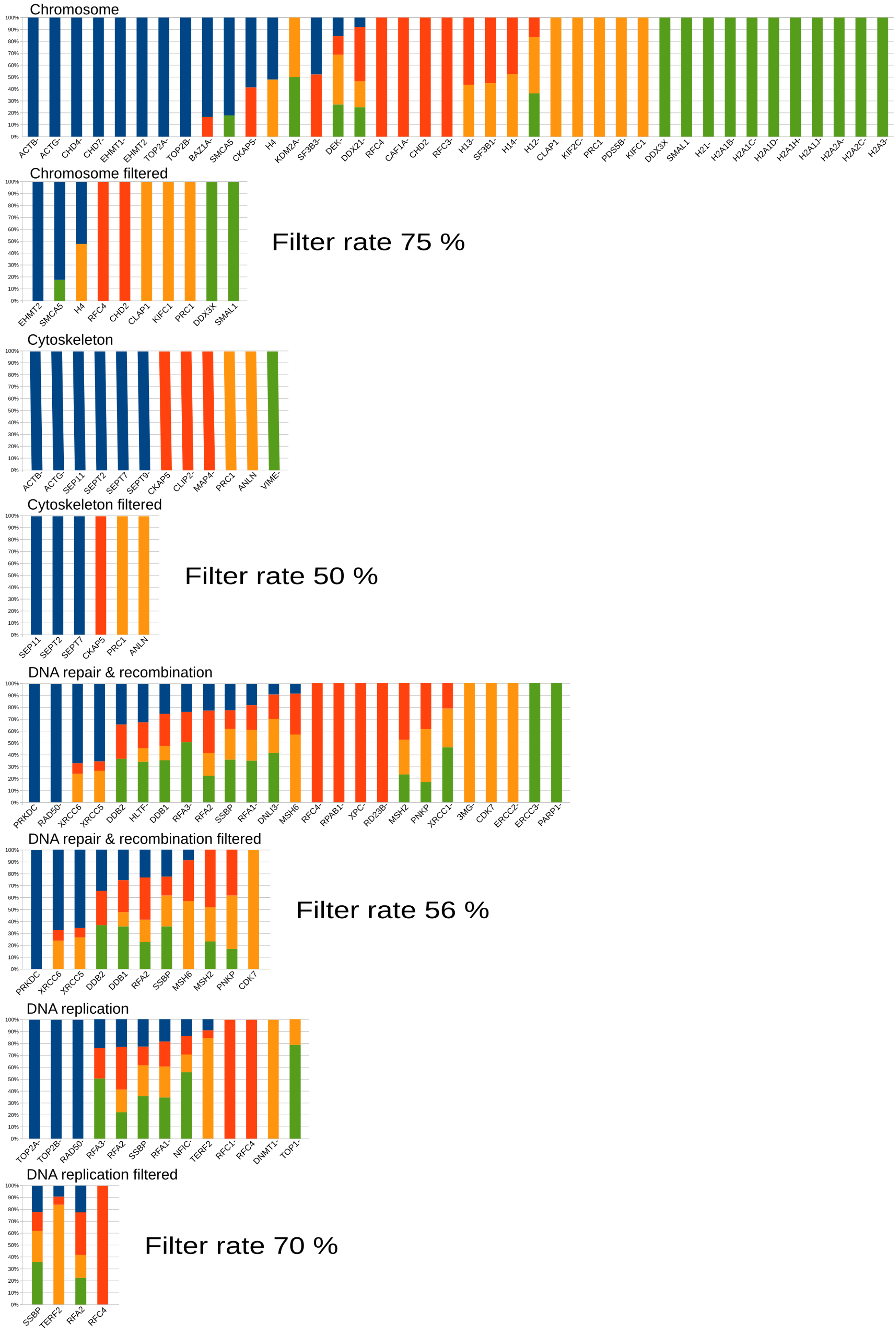

Fig. S5: Distribution of the proteins differentially distributed into one or more bands after purification with either the control or the DNA ends phase. The proteins accounted for in this diagram were identified at least 3 times over the 6 experiments. Proteins were sorted by their relative abundance in the 720, 480, 240 kDa and common bands, in that order. For each functional category, the bottom histogram corresponds to the same data as for the top one, but after filtering applied (see *Materials and Methods*). The name of the proteins that are filtered out is followed by a dash. Band color code (see Fig. 3A of the main text): blue, 720 kDa; red, 480 kDa; yellow, 240 kDa; green: common. (follows in Fig. S6).

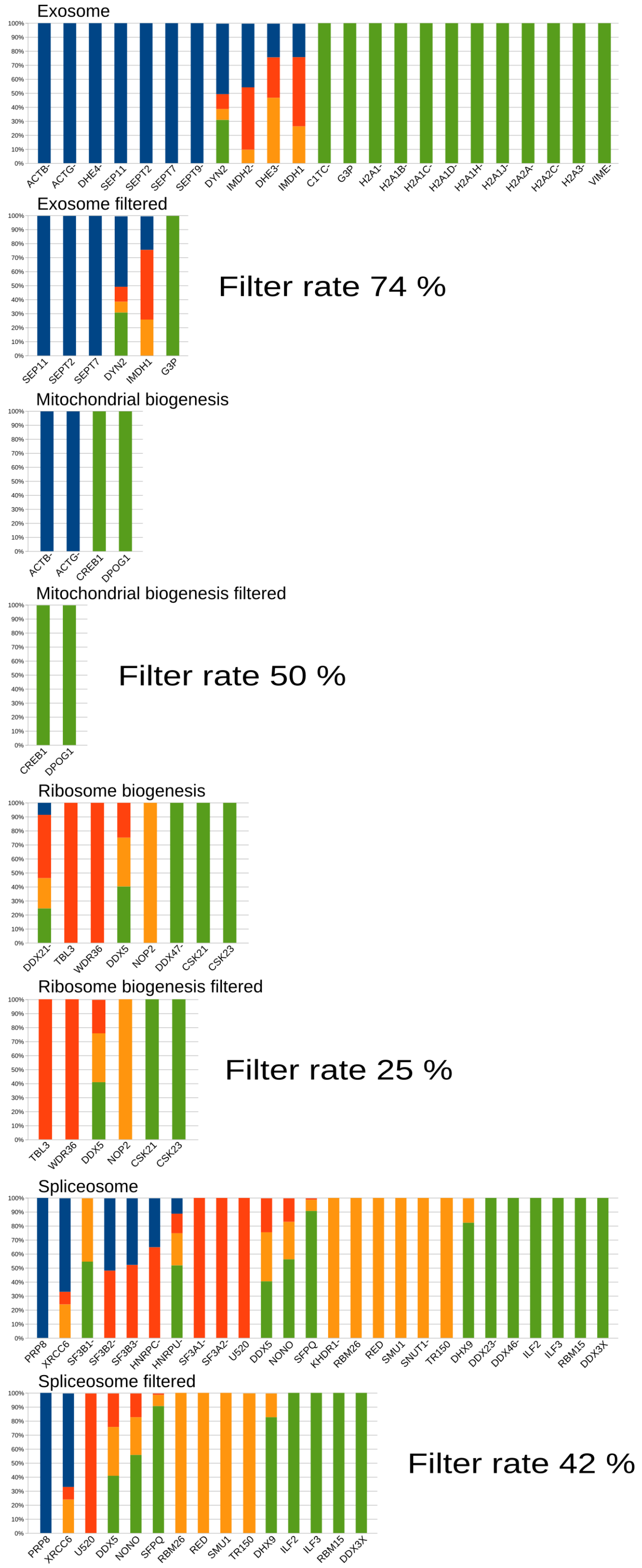

Fig. S6: Distribution of the proteins differentially distributed into one or more bands after purification with either the control or the DNA ends phase (continued from Fig. S5, follows in Fig. S7).

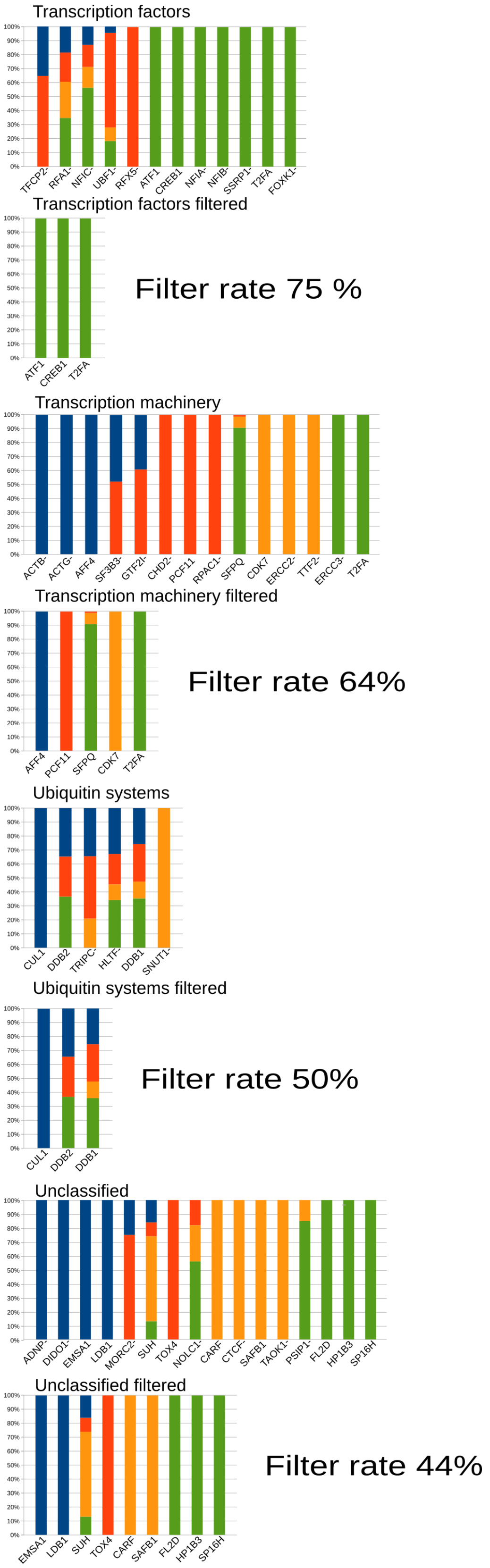

Fig. S7: Distribution of the proteins differentially distributed into one or more bands after purification with either the control or the DNA ends phase (continued from Fig. S6).

Table S8: Characteristics of the proteins identified as exclusively specific of the DNA ends phase. The proteins are listed along with their relative quantifications in the various native gel bands (mean of the relative quantifications for six [DNA ends phase *vs* control phase] paired experiments along with the standard deviation in parentheses), their related functional classes and their involvement in the XRCC5 or XRCC6 or DNA-PKcs interactomes (Biogrid data). Proteins not purified in a given band are described as '–'.

	Presence in the various bands					
Protein	common	240 kDa	480 kDa	720 kDa	Func. class	Interactions
AFF4<br>AF4/FMR2 family member 4	–	–	–	100 (0)	trm	-
ANLN<br>Actin-binding protein anillin	–	100 (0)	–	–	cyt	-
ATF1<br>Cyclic AMP-dependent transcription factor ATF-1	100 (0)	–	–	–	trf	-
CARF<br>CDKN2A-interacting protein	–	100 (0)	–	–	uncl	-
CDK7<br>Cyclin-dependent kinase 7	–	100 (0)	–	–	dnarr;trm	-
CHD2<br>Chromodomain-helicase-DNA-binding protein 2	–	–	100 (0)	–	chr	-
CKAP5<br>Cytoskeleton-associated protein 5	–	–	43 (27)	57 (23)	cyt	-
CLAP1<br>CLIP-associating protein 1	–	100 (0)	–	–	chr	-
CREB1<br>Cyclic AMP-responsive element-binding protein 1	100 (0)	–	–	–	mit;trf	-
CSK21<br>Casein kinase II subunit alpha	100 (0)	–	–	–	rib	X5,X6,PK
CSK23<br>Casein kinase II subunit alpha 3	100 (0)	–	–	–	rib	X5,X6,PK
CUL1<br>Cullin-1	–	–	–	100 (0)	ub	-
DDB1*<br>DNA damage-binding protein 1	36 (12)	12 (5)	27 (11)	25 (18)	dnarr;ub	-
DDB2<br>DNA damage-binding protein 2	37 (9)	–	27 (19)	36 (19)	dnarr;ub	X6
DDX3X<br>ATP-dependent RNA helicase DDX3X	100 (0)	–	–	–	chr;spli	-
DDX5<br>Probable ATP-dependent RNA helicase DDX5	41 (24)	35 (14)	24 (11)	–	rib;spli	PK
DHX9<br>ATP-dependent RNA helicase A	83 (10)	17 (2)	–	–	spli	X5,X6,PK
DPOG1<br>DNA polymerase subunit gamma-1	100 (0)	–	–	–	mit	-
DYN2*<br>Dynamin-2	31 (12)	8 (3)	10 (6)	51 (11)	exo	-
EHMT2<br>Histone-lysine N-methyltransferase EHMT2	–	–	–	100 (0)	chr	-
EMSA1<br>ELM2 and SANT domain-containing protein 1	–	–	–	100 (0)	uncl	-
FL2D<br>Pre-mRNA-splicing regulator WTAP	100 (0)	–	–	–	uncl	-
G3P<br>Glyceraldehyde-3-phosphate dehydrogenase	100 (0)	–	–	–	exo	-
H4<br>Histone H4	–	48 (15)	–	52 (24)	chr	-
HP1B3<br>Heterochromatin protein 1-binding protein 3	100 (0)	–	–	–	uncl	-
ILF2	100 (0)	–	–	–	spli	X5,X6,PK

(follows)

Table S8: **Characteristics of the proteins identified as DNA ends phase-specific (followed)**

Protein	Presence in the various bands common	240 kDa	480 kDa	720 kDa	Func. class	Interactions
Interleukin enhancer-binding factor 2						
ILF3	100 (0)	–	–	–	spli	PK
Interleukin enhancer-binding factor 3						
IMDH1*	–	26 (12)	50 (26)	24 (16)	exo	X6
Inosine-5'-monophosphate dehydrogenase 1						
KIFC1	–	100 (0)	–	–	chr	-
Kinesin-like protein KIFC1						
LDB1	–	–	–	100 (0)	uncl	-
LIM domain-binding protein 1						
MSH2	23 (6)	30 (35)	47 (27)	–	dnarr	X5,X6
DNA mismatch repair protein Msh2						
MSH6	–	62 (18)	38 (9)	9 (7)	dnarr	-
DNA mismatch repair protein Msh6						
NONO	56 (26)	27 (11)	17 (13)	–	spli	-
Non-POU domain-containing octamer-binding protein						
NOP2	–	100 (0)	–	–	rib	-
Probable 28S rRNA (cytosine(4447)-C(5))-methyltransferase						
PCF11	–	–	100 (0)	–	trm	-
Pre-mRNA cleavage complex 2 protein Pcf11						
PNKP	10 (1)	48 (11)	42 (11)	–	dnarr	X5,X6,PK
Bifunctional polynucleotide phosphatase/kinase						
PRC1	–	100 (0)	–	–	chr;cyt	-
Protein regulator of cytokinesis 1						
PRKDC	–	–	–	100 (0)	dnarr	X5,X6
DNA-dependent protein kinase catalytic subunit						
PRP8	–	–	–	100 (0)	spli	PK
Pre-mRNA-processing-splicing factor 8						
RBM15	100 (0)	–	–	–	spli	-
Putative RNA-binding protein 15						
RBM26	–	100 (0)	–	–	spli	-
RNA-binding protein 26						
RED	–	100 (11)	–	–	spli	-
IK						
RFA2*	23 (6)	19 (4)	35 (34)	23 (4)	dnarr	X5,X6,PK
Replication protein A 32 kDa subunit						
RFC4	–	–	100 (0)	–	chr;dnarep	-
Replication factor C subunit 4						
SAFB1	–	100 (0)	–	–	uncl	-
Scaffold attachment factor B1						
SEP11	–	–	–	100 (0)	cyt;exo	-
Septin-11						
SEPT2	–	–	–	100 (0)	cyt;exo	-
Septin-2						
SEPT7	–	–	–	100 (0)	cyt;exo	-
Septin-7						
SFPQ	91 (5)	8 (4)	1 (1)	0 (0)	spli;trm	-
Splicing factor, proline- and glutamine-rich						
SMAL1	100 (0)	–	–	–	chr	-
SWI/SNF-related matrix-associated actin-dependent regulator of chromatin subfamily A-like protein 1						
SMCA5	18 (4)	–	–	82 (1)	chr	X5,PK
SWI/SNF-related matrix-associated actin-dependent regulator of chromatin subfamily A member 5						
SMU1	–	100 (0)	–	–	spli	-
WD40 repeat-containing protein SMU1						
SP16H	100 (0)	–	–	–	uncl	X5,X6
FACT complex subunit SPT16						
SSBP*	36 (22)	26 (7)	16 (6)	22 (10)	dnarr;dnarep	-
Single-stranded DNA-binding protein, mitochondrial						

(follows)

Table S8: **Characteristics of the proteins identified as DNA ends phase-specific (followed)**

	Presence in the various bands					
Protein	common	240 kDa	480 kDa	720 kDa	Func. class	Interactions
SUH* Recombining binding protein suppressor of hairless	13 (4)	61 (15)	10 (2)	16 (7)	uncl	-
T2FA General transcription factor IIF subunit 1	100 (0)	–	–	–	trf;trm	-
TBL3 Transducin beta-like protein 3	–	–	100 (0)	–	rib	-
TERF2 Telomeric repeat-binding factor 2	–	85 (6)	7 (1)	9 (6)	dnarep	X5,X6
TOX4 TOX high mobility group box family member 4	–	–	100 (0)	–	uncl	-
TR150 Thyroid hormone receptor-associated protein 3	–	100 (0)	–	–	spli	-
U520 U5 small nuclear ribonucleoprotein 200 kDa helicase	–	–	100 (0)	–	spli	-
WDR36 WD repeat-containing protein 36	–	–	100 (0)	–	rib	-
XRCC5* X-ray repair cross-complementing protein 5	–	27 (18)	8 (7)	66 (24)	dnarr	X6,PK
XRCC6* X-ray repair cross-complementing protein 6	–	24 (16)	9 (7)	67 (22)	dnarr;spli	X5,PK

Legend. chr: chromosome, cyt: cytoskeleton, dnarr: DNA repair & recombination, dnarep: DNA replication, exo: exosome, mit: mitochondrial biogenesis, rib: ribosome biogenesis, spli: spliceosome, trf: transcription factors, trm: transcription machinery, ub: tbiquitin systems, uncl: unclassified. X5: XRCC5 (Ku80), X6: XRCC6 (Ku70), PK: PRKDC (DNA-PKcs). The asterisk next to the protein name indicates that the protein was found to be part of the core DNA ends proteome, as described in Fig. 3A of the main text. Parenthesized numerical values indicate the standard deviation of the associated percentage value.

Table S9: DNA ends *vs* control phase specificity of the proteins in the various bands as the mean of the ratios $\left[\frac{M_i}{M_i+B_i} \cdot 100\right]$ for six [DNA ends phase *vs* control phase] paired experiments (see *Materials and Methods* ; standard deviation in parentheses). Same protein set as in the previous table ; the same information is reproduced for convenience. Proteins not purified in a given band are described as '–'.

	Presence in the various bands					
Protein	common	240 kDa	480 kDa	720 kDa	Func. class	Interactions
AFF4<br>AF4/FMR2 family member 4	–	–	–	100 (0)	trm	-
ANLN<br>Actin-binding protein anillin	–	100 (0)	–	–	cyt	-
ATF1<br>Cyclic AMP-dependent transcription factor ATF-1	100 (0)	–	–	–	trf	-
CARF<br>CDKN2A-interacting protein	–	100 (0)	–	–	uncl	-
CDK7<br>Cyclin-dependent kinase 7	–	92 (15)	–	–	dnarr;trm	-
CHD2<br>Chromodomain-helicase-DNA-binding protein 2	–	–	100 (0)	–	chr	-
CKAP5<br>Cytoskeleton-associated protein 5	–	–	100 (0)	100 (0)	cyt	-
CLAP1<br>CLIP-associating protein 1	–	99 (2)	–	–	chr	-
CREB1<br>Cyclic AMP-responsive element-binding protein 1	100 (0)	–	–	–	mit;trf	-
CSK21<br>Casein kinase II subunit alpha	100 (0)	–	–	–	rib	X5,X6,PK
CSK23<br>Casein kinase II subunit alpha 3	100 (0)	–	–	–	rib	X5,X6,PK
CUL1<br>Cullin-1	–	–	–	100 (0)	ub	-
DDB1*<br>DNA damage-binding protein 1	90 (10)	68 (24)	90 (17)	92 (19)	dnarr;ub	-
DDB2<br>DNA damage-binding protein 2	97 (5)	–	89 (16)	100 (0)	dnarr;ub	X6
DDX3X<br>ATP-dependent RNA helicase DDX3X	100 (0)	–	–	–	chr;spli	-
DDX5<br>Probable ATP-dependent RNA helicase DDX5	100 (0)	100 (0)	100 (0)	–	rib;spli	PK
DHX9<br>ATP-dependent RNA helicase A	92 (11)	100 (0)	–	–	spli	X5,X6,PK
DPOG1<br>DNA polymerase subunit gamma-1	100 (0)	–	–	–	mit	-
DYN2*<br>Dynamin-2	100 (0)	100 (0)	100 (0)	100 (0)	exo	-
EHMT2<br>Histone-lysine N-methyltransferase EHMT2	–	–	–	100 (0)	chr	-
EMSA1<br>ELM2 and SANT domain-containing protein 1	–	–	–	94 (11)	uncl	-
FL2D<br>Pre-mRNA-splicing regulator WTAP	98 (5)	–	–	–	uncl	-
G3P<br>Glyceraldehyde-3-phosphate dehydrogenase	100 (0)	–	–	–	exo	-
H4<br>Histone H4	–	56 (16)	–	100 (0)	chr	-
HP1B3<br>Heterochromatin protein 1-binding protein 3	100 (0)	–	–	–	uncl	-
ILF2	100 (0)	–	–	–	spli	X5,X6,PK

(follows)

Table S9: **DNA ends *vs* control phase specificity of the proteins in the various bands as the mean of the ratios** $\left[\frac{M_i}{M_i+B_i}\cdot 100\right]$ **(followed).**

	Presence in the various bands					
Protein	common	240 kDa	480 kDa	720 kDa	Func. class	Interactions
Interleukin enhancer-binding factor 2						
ILF3	100 (0)	–	–	–	spli	PK
Interleukin enhancer-binding factor 3						
IMDH1*	–	100 (0)	100 (0)	90 (20)	exo	X6
Inosine-5'-monophosphate dehydrogenase 1						
KIFC1	–	100 (0)	–	–	chr	-
Kinesin-like protein KIFC1						
LDB1	–	–	–	90 (15)	uncl	-
LIM domain-binding protein 1						
MSH2	100 (0)	90 (17)	96 (8)	–	dnarr	X5,X6
DNA mismatch repair protein Msh2						
MSH6	–	91 (11)	100 (0)	100 (0)	dnarr	-
DNA mismatch repair protein Msh6						
NONO	92 (17)	82 (32)	100 (0)	–	spli	-
Non-POU domain-containing octamer-binding protein						
NOP2	–	100 (0)	–	–	rib	-
Probable 28S rRNA (cytosine(4447)-C(5))-methyltransferase						
PCF11	–	–	100 (0)	–	trm	-
Pre-mRNA cleavage complex 2 protein Pcf11						
PNKP	100 (0)	50 (19)	56 (20)	–	dnarr	X5,X6,PK
Bifunctional polynucleotide phosphatase/kinase						
PRC1	–	95 (6)	–	–	chr;cyt	-
Protein regulator of cytokinesis 1						
PRKDC	–	–	–	91 (5)	dnarr	X5,X6
DNA-dependent protein kinase catalytic subunit						
PRP8	–	–	–	93 (15)	spli	PK
Pre-mRNA-processing-splicing factor 8						
RBM15	100 (0)	–	–	–	spli	-
Putative RNA-binding protein 15						
RBM26	–	100 (0)	–	–	spli	-
RNA-binding protein 26						
RED	–	100 (0)	–	–	spli	-
IK						
RFA2*	90 (14)	83 (24)	72 (28)	75 (18)	dnarr	X5,X6,PK
Replication protein A 32 kDa subunit						
RFC4	–	–	100 (0)	–	chr;dnarep	-
Replication factor C subunit 4						
SAFB1	–	100 (0)	–	–	uncl	-
Scaffold attachment factor B1						
SEP11	–	–	–	100 (0)	cyt;exo	-
Septin-11						
SEPT2	–	–	–	100 (0)	cyt;exo	-
Septin-2						
SEPT7	–	–	–	100 (0)	cyt;exo	-
Septin-7						
SFPQ	89 (9)	87 (8)	91 (12)	84 (32)	spli;trm	-
Splicing factor, proline- and glutamine-rich						
SMAL1	91 (13)	–	–	–	chr	-
SWI/SNF-related matrix-associated actin-dependent regulator of chromatin subfamily A-like protein 1						
SMCA5	100 (0)	–	–	61 (10)	chr	X5,PK
SWI/SNF-related matrix-associated actin-dependent regulator of chromatin subfamily A member 5						
SMU1	–	92 (10)	–	–	spli	-
WD40 repeat-containing protein SMU1						
SP16H	95 (8)	–	–	–	uncl	X5,X6
FACT complex subunit SPT16						

(follows)

Table S9: **DNA ends *vs* control phase specificity of the proteins in the various bands as the mean of the ratios $\left[\frac{M_i}{M_i+B_i} \cdot 100\right]$ (followed).**

	Presence in the various bands					
Protein	common	240 kDa	480 kDa	720 kDa	Func. class	Interactions
SSBP* Single-stranded DNA-binding protein, mitochondrial	81 (17)	75 (10)	87 (17)	90 (15)	dnarr;dnarep	-
SUH* Recombining binding protein suppressor of hairless	93 (13)	56 (13)	75 (25)	82 (24)	uncl	-
T2FA General transcription factor IIF subunit 1	100 (0)	–	–	–	trf;trm	-
TBL3 Transducin beta-like protein 3	–	–	100 (0)	–	rib	-
TERF2 Telomeric repeat-binding factor 2	–	79 (4)	86 (24)	100 (0)	dnarep	X5,X6
TOX4 TOX high mobility group box family member 4	–	–	100 (0)	–	uncl	-
TR150 Thyroid hormone receptor-associated protein 3	–	100 (0)	–	–	spli	-
U520 U5 small nuclear ribonucleoprotein 200 kDa helicase	–	–	100 (0)	–	spli	-
WDR36 WD repeat-containing protein 36	–	–	93 (9)	–	rib	-
XRCC5* X-ray repair cross-complementing protein 5	–	95 (7)	97 (2)	94 (4)	dnarr	X6,PK
XRCC6* X-ray repair cross-complementing protein 6	–	97 (3)	94 (10)	94 (5)	dnarr;spli	X5,PK

Legend. chr: chromosome, cyt: cytoskeleton, dnarr: DNA repair & recombination, dnarep: DNA replication, exo: exosome, mit: mitochondrial biogenesis, rib: ribosome biogenesis, spli: spliceosome, trf: transcription factors, trm: transcription machinery, ub: tbiquitin systems, uncl: unclassified. X5: XRCC5 (Ku80), X6: XRCC6 (Ku70), PK: PRKDC (DNA-PKcs). The asterisk next to the protein name indicates that the protein was found to be part of the core DNA ends proteome, as described in Fig. 3A of the main text.

From a data mining standpoint, the home-made software is released as Free Software and made available as source code, for GNU/Linux and Mac OS X, or as a package for the MS-Windows platform.

Material available on the internet

The reader are granted access to a perennial institutional (CNRS) webdav-based repository. The material is accessed using the following link:

`https://mycore.core-cloud.net/public.php?service=files&t=7caf98c63238d779beb6e567ee579e34`

No credentials are needed to get access to the following material:

* `User Manual`
  This User Manual briefly documents the scientific project, the database structure and the workings of the DNA-Ends-Proteome program. A crash course in SQL (structured query language) is provided to make the most out of the database (see below);

* `DNA-Ends-Proteome program`
  This program is intended to allow the user to perform interrogations on the DNA-Ends-Proteome.db database;

* `DNA-Ends-Proteome.db database`
  This database stores all the tandem mass-based protein identification data.
