# Supplementary material for: The human DNA ends proteome uncovers an unexpected entanglement of functional pathways
Source: Nucleic Acids Res. 2016 Feb 25;44(10):4721–33. doi: 10.1093/nar/gkw121 (PMC4889927; doi:10.1093/nar/gkw121)
Supplement: SUPPLEMENTARY DATA [file supp_44_10_4721__index.html]

The human DNA ends proteome uncovers an unexpected entanglement of functional pathways — The human DNA ends proteome uncovers an unexpected entanglement of functional pathways — SUPPLEMENTARY DATA 

# The human DNA ends proteome uncovers an unexpected entanglement of functional pathways

## SUPPLEMENTARY DATA

- SUPPLEMENTARY DATA
- SUPPLEMENTARY DATA
